# Evidence for Cardiac Phase‐Linked Perception of Heartbeats

**DOI:** 10.1111/psyp.70346

**Published:** 2026-06-25

**Authors:** Ren Palmer, Davide Morelli, David Plans, Jennifer Todd, Jane Aspell, Mateo Leganes, Geoffrey Bird, Jennifer Murphy

**Affiliations:** ^1^ School of Psychology University of Surrey Surrey UK; ^2^ Department of Engineering Science Institute of Biomedical Engineering, University of Oxford Oxford UK; ^3^ Department of Psychology Royal Holloway University of London Surrey UK; ^4^ School of Psychology, Sport and Sensory Sciences Anglia Ruskin University Cambridge UK; ^5^ Centre for Psychological Medicine Perdana University Kuala Lumpur Malaysia; ^6^ Louvain Experimental Psychopathology Research Group (LEP) Psychological Science Research Institute Louvain‐la‐Neuve Belgium; ^7^ Developmental Psychopathology Department, Psychology School University of Amsterdam Amsterdam the Netherlands; ^8^ Department of Experimental Psychology University of Oxford Oxford UK; ^9^ Centre for Research in Autism and Education, Institute of Education University College London London UK

## Abstract

Commonly used methods for assessing cardiac interoceptive accuracy have been criticized for assuming that all individuals perceive their heartbeat at the same delay following contraction of the heart, despite evidence for notable variability across individuals. However, it remains unclear whether some individuals perceive their heartbeat at a particular phase of their cardiac cycle—that is, at a relative point in the cycle that may vary in absolute timing depending on heart rate—rather than at a specific delay. Identification of all heartbeat perceivers is critical for accurate measurement of cardiac interoceptive accuracy; individual differences in which are theorized to play a role in several aspects of higher‐order cognition as well as health and wellbeing. In the current study, data from 526 participants who completed the Phase Adjustment Task (PAT) as a measure of cardiac interoceptive accuracy were examined. In this task, participants are asked to adjust a virtual dial until tones appear synchronous with their heartbeats. Data were analyzed using a novel framework that allows differentiation between delay‐based and phase‐based response patterns. Of 76 interoceptive individuals identified, 21% (*N* = 16) demonstrated response patterns consistent only with phase‐based responding. These novel findings challenge current assumptions regarding individual differences in the perception of heartbeats, and suggest that many commonly used measures may underestimate the true proportion of heartbeat perceivers.

## Impact Statement

This work challenges the longstanding assumption that all individuals perceive heartbeats at a fixed delay following heartbeat contraction—a core assumption of many cardiac interoceptive accuracy tasks. Applying a novel analytic approach to data from 500+ participants, we found that 21% of individuals exhibited non‐random responding consistent with a phase‐based response pattern. These findings challenge core assumptions in the field and suggest that existing measures may systematically underestimate the proportion of heartbeat perceivers.

Interoception—the processing of internal bodily signals—has garnered increasing attention in recent years (Khalsa and Lapidus 2016). In particular, cardiac interoceptive accuracy, the ability to perceive one's heartbeat, has become a focus of research, with much theoretical and empirical work linking individual differences in cardiac interoceptive accuracy to a range of mental health conditions and higher‐order cognitive functions (Brewer et al. 2021). However, despite growing interest, there is increasing concern about the validity of commonly used measures to assess individual differences in cardiac interoceptive accuracy (Adams et al. 2022; Desmedt et al. 2023).

Traditionally, studies have primarily relied on the Heartbeat Counting Task (HCT; Dale and Anderson 1978; Schandry 1981). In the HCT, participants are required to count the number of heartbeats that occur over a series of fixed time intervals while objective heart rate is recorded. The subjective and objective counts are then compared to determine accuracy. Despite widespread use, a large body of evidence challenges the validity of this task as a measure of perceptual ability. Such work suggests that participants may rely on estimation strategies, including knowledge of their own heart rate and time estimation, to achieve good performance in the absence of an ability to perceive heartbeats (Desmedt et al. 2018; Murphy et al. 2018; see Brener and Ring 2016). As a result, the HCT has come under increasing criticism, and attention has shifted toward alternative measures (Brener and Ring 2016; Desmedt et al. 2023).

Multisensory integration tasks involve asking participants to determine whether an external stimulus (auditory, visual or tactile) is presented synchronously or asynchronously with their heartbeat (Brener and Ring 2016). In the most commonly used variant—the two‐alternative forced choice heartbeat detection task (2AFC‐HDT; first proposed by Whitehead et al. 1977)—‘synchronous’ tones are typically presented after a delay of ~200–300 ms following the R‐wave (with ‘asynchronous’ tones presented after a delay of ~500 ms) and accuracy is determined by the number of ‘correct’ synchronous judgments (e.g., Garfinkel et al. 2016; Herman et al. 2019; Kleckner et al. 2015). This implementation and scoring is based on the assumption that most individuals perceive heartbeats at a delay of approximately 100–300 ms following the R‐wave (Betka et al. 2020; Brener and Kluvitse 1988; Yates et al. 1985). However, there are limitations to this approach. First, evidence suggests there is significant variation in the delay at which tones are perceived as synchronous with heartbeats, ranging from 0 to 400 ms (Clemens 1984; Yates et al. 1985). These individual differences are so pronounced that some participants perceive synchrony with the ‘asynchronous’ tone (Brener et al. 1993; Wittkamp et al. 2018). Notably this is not a negligible proportion of participants, and has been reported across several studies (e.g., Brener et al. 1993; Wittkamp et al. 2018). To account for this, some studies use alternative scoring strategies where a consistent selection of either predefined synchronous or asynchronous tones is taken as evidence for heartbeat perception (Brener et al. 1993). Second, studies vary widely in their administration. Indeed, although typically presented at 200–300 ms following the R‐wave, synchronous presentations have ranged from 0 to 300 ms across studies (e.g., Gardner et al. 1990; Lyyra and Parviainen 2018). In other variants, tones are presented at a particular proportion of the inter‐beat interval (IBI; Knoll and Hodapp 1992; Lyyra and Parviainen 2018; Mul et al. 2018; Salomon et al. 2016), based on the assumption that participants should perceive heartbeat‐tone synchrony at systole, rather than diastole (see also Smith et al. 2020). However, regardless of implementation and scoring of the 2AFC, it is well acknowledged that the 2AFC HDT may produce false negatives (i.e., individuals incorrectly judged to be unable to perceive their heartbeats; Brener and Ring 2016). This is because individuals who perceive heartbeat‐tone synchrony at a point that is equidistant to presented synchronous and asynchronous delays would likely respond randomly, as neither delay aligns with their perception of heartbeat‐tone synchrony (Brener and Ring 2016; Adams et al. 2022, 2026). Similarly, if individuals perceive their heartbeats at a point during diastole, as opposed to systole, they would be routinely misclassified by existing procedures that assume stimuli are perceived at systole, even if those procedures present tones at varying proportions of the IBI.

Due to these limitations, focus has shifted toward tasks that do not make assumptions about the time at which an individual should perceive a tone to be synchronous with their heartbeat (Desmedt et al. 2023). Older approaches using such methods include the six‐alternative forced choice (6AFC) HDT and Method of Constant Stimuli (MCS), where tones are presented at multiple fixed delays following the R‐wave (e.g., 0–500 ms), participants select the delays at which tones are perceived as synchronous with heartbeats, and accuracy is inferred from the consistency of participants' selections over trials (Brener et al. 1993; Yates et al. 1985). Consistency is typically assessed using chi‐square analyses (χ^2^ test), testing for non‐random responding across all presented delays, or the interquartile range (IQR) of the distribution of selected delays, where smaller IQRs imply a narrower window of heartbeat‐tone synchrony (Brener and Ring 2016). Using the χ^2^ test for both the 2AFC, 6AFC and MCS (making no assumption of when heartbeats are perceived for the 2AFC), typically more participants show above chance performance on the 6AFC (50%) and MCS (54%) when compared to the 2AFC (33%; Brener et al. 1993). Such a pattern is generally assumed to reflect that some individuals will be falsely deemed ‘non‐interoceptive^1^’ (or as ‘non‐heartbeat detectors’; Brener et al. 1993) on the 2AFC HDT, because the delays at which tones are presented do not align with their perception of heartbeat‐tone synchrony.

More recent implementations of the MCS/6AFC task have moved away from the use of the χ^2^ test and have begun to assess performance based on the IQR (e.g., Moffatt et al. 2024; Ring and Brener 2018; Ruisch et al. 2023; see for discussion Wiens and Palmer 2001). While this approach does not make an assumption about the timing of heartbeat perception, use of the IQR as a scoring method rests on the assumption that all individuals perceive heartbeat‐tone synchrony at a specific, single, delay following the R‐wave. Indeed, although reportedly not common, it has been noted in literature reviews that some individuals select multiple delays as synchronous on the MCS/6AFC (Brener and Ring 2016). Although these individuals' selections would likely be non‐random (as determined by the χ^2^ test), a multiple delay response pattern would result in a larger IQR, as selected delays would be more distributed than if they selected a single delay. The selection of multiple delays as synchronous by some participants suggests the assumption that all individuals perceive heartbeat‐tone synchrony at a specific, and single, delay following the R‐wave is flawed. This has relevance for modern approaches to the scoring of the heartbeat tapping task (HTT; Körmendi et al. 2022; Smith et al. 2020, 2021). The HTT requires participants to tap their finger or press down on a key when they perceive heartbeats (Hamano 1980; Kleinman 1970; Ludwick‐Rosenthal and Neufeld 1985; McFarland 1975). This is sometimes completed under different instructions (e.g., guessing or no guessing conditions; Smith et al. 2020). Previous approaches to scoring the HTT, much like the HCT, can result in non‐interoceptive individuals being deemed to be interoceptive due to informed guessing, particularly when accuracy is determined solely by the difference between the number of real heartbeats vs. taps. Newer approaches to HTT scoring avoid this issue, typically using one of two different methods (Smith et al. 2020, 2021). The first method is conceptually similar to the IQR method described above. In this method, participants' tapping responses are coded as negative or positive values with respect to the closest heartbeat. Like the IQR, the variability in responses is quantified and then compared to random responding after conversion to Z‐scores. The second uses a Bayesian computational model to calculate the consistency of participants' selections over trials in a manner that is similar to the χ^2^ approach to scoring of the MCS/6AFC. In the second method, taps are defined as “correct” when they occur within a predefined window, typically one that broadly corresponds with systole after approximate correction for pulse arrival time (PTT^2^) and motor responses (e.g., 350–650 ms or R‐peak +200 ms (for PTT correction) to 50% of the inter‐beat interval (IBI); Körmendi et al. 2022; Smith et al. 2020). Although a large “correct” response window is provided, this method—like the IQR scoring approach—assumes that all individuals perceive heartbeat‐tone synchrony within a specific range of delays following the R‐wave, or at a specific point in the cardiac cycle (e.g., systole).

Exactly why some participants display non‐random responding and yet perceive synchrony at multiple delays on the MCS/6AFC remains unclear. Previous explanations include variability in the locations from which heartbeats are perceived over trials (see Brener and Ring 2016), or it may be that differences in the duration of the heartbeat stimulus vs. presented tones produces temporal overlap in their sensory representations (i.e., trace effects). Although these potential explanations remain to be empirically examined, an alternative possibility is that some participants perceive heartbeat‐tone synchrony at a particular phase of their cardiac cycle, rather than at a specific delay (where the term phase refers to a specific point in the cardiac cycle rather than a specific physiological process such as systole). With respect to the delay at which heartbeats are perceived, although the consistent duration of systole of approximately 350 ms regardless of heart rate (Wallace et al. 1963; Weissler et al. 1968; Betts 2013) might be expected to result in heartbeat perception within previously used stimulus windows (e.g., 0–400 ms) under the (incorrect; Brener and Ring 2016; Al et al. 2020) assumption that the heartbeat is only perceived during systole, there is no reason to expect that the stable duration of systole would produce the same selected *delay* over trials (e.g., a consistent selection of 300 ms rather than selections between 0 and 350 ms on the MCS/6AFC HDT). Indeed, as the temporal window of discrimination of heartbeat‐tone synchrony is unknown and could vary between and within individuals (just as the perceptual timing of two exteroceptive stimuli may also vary between and within participants), there are multiple possible explanations for both (1) heartbeats being perceived at different delays following the r‐wave across participants, and (2) at multiple timepoints within the same participant, which may, or may not, correspond to a consistent phase of their cardiac cycle.

Importantly, if some individuals select delays that correspond to a consistent phase of the cardiac cycle rather than a fixed delay after the R‐wave, these individuals would select multiple delays on tasks such as the MCS and 6AFC when there is large variability in heart rate over trials. Under stable heart rate conditions, a fixed delay (e.g., 250 ms) will coincide with a consistent phase of the cardiac cycle (e.g., 250 ms represents 25% of a 1000 ms IBI). However, when heart rate fluctuates, the same phase of the cardiac cycle maps onto multiple delays; for example, 25% of a 1000 ms IBI corresponds to a delay of 250 ms, but a delay of 125 ms with a 500 ms IBI. As a consequence, where heart rate varies over trials—not unlikely for lengthy tasks such as the MCS/6AFC (Shaffer and Ginsberg 2017)—individuals making phase‐based responses would not perceive tones as synchronous with their heartbeat at the same delay over trials, resulting in the selection of multiple delays on the MCS/6AFC (Brener and Ring 2016; and potentially taps outside of predefined windows on the HTT depending on preferred phases). Thus, phase‐based responses would increase the IQR of selected delays on the MCS/6AFC and reduce the consistency of participants' tapping responses on the HTT, making performance appear less accurate, even when responding is not random (Körmendi et al. 2022; Moffatt et al. 2024; Ring and Brener 2018; Ruisch et al. 2023; Smith et al. 2021). Indeed, if a subset of individuals use phase‐based strategies, these individuals would also likely be deemed non‐interoceptive on the 2AFC task, which (alongside individual differences in the delay at which individuals perceive heartbeat tone synchrony; Adams et al. 2026; Brener and Ring 2016) may also contribute toward the lower than expected correspondence between the MCS/6AFC tasks and the 2AFC HDT (Brener et al. 1993).

To our knowledge, no study has yet examined whether a subset of individuals perceive heartbeat‐tone synchrony at a particular phase of their cardiac cycle, rather than a specific delay (Adams et al. 2026). As such, this study aimed to explore this question, using a measure of cardiac interoceptive accuracy—the Phase Adjustment Task (PAT; Plans et al. 2021)—that makes no assumption regarding the timing at which individuals perceive heartbeat‐tone synchrony. In the PAT, participants are presented with a series of tones at their heartbeat frequency, but out of phase with heartbeats. They are asked to adjust a virtual dial until they perceive the tones to be synchronous with their heartbeats. As the starting phase is random across trials, interoceptive accuracy is inferred from the consistency of responses across trials. In this study, we utilized the revised scoring framework introduced in Palmer et al. 2026, in a sample of 526 participants. By modeling each participant's response pattern relative to both absolute delays and relative phase as a function of IBI, this approach provides the first analytical framework capable of quantifying and distinguishing between delay‐based and phase‐based responding (PAT 2.0; Palmer et al. 2026). Based on the aforementioned evidence of multiple delay response patterns on the MCS/6AFC, we predicted that at least some interoceptive participants would show phase‐based non‐random responding. However, as this is the first study to quantify phase‐based responding, we made no prediction regarding the proportion of individuals in which this would occur.

## 1 Methods

### 1.1 Ethical Approval and Data Availability

Data were drawn from several datasets, each of which obtained ethical approval from the relevant local ethics committees at Royal Holloway University of London, Anglia Ruskin University, and Rutgers University. All participants gave informed consent prior to participation and were fully debriefed on completion. Participation was incentivized through monetary compensation and course credits for Psychology students.

### 1.2 Participants

Data were pooled from every study that used the PAT to date. In total, six datasets were used: two collected in a laboratory under supervised conditions and four completed remotely (for details see Supplement S5). These datasets were merged given evidence that PAT performance does not significantly differ between lab‐based and remote administration (Palmer et al. 2026; Spooner, Bird, Clemente, et al. 2024). 380 participants completed the original PAT application (Plans et al. 2021) and 146 completed the new application (PAT 2.0; Plans et al. 2021, under review). Although the new application includes some new features (e.g., an anti‐cheat mechanism, two additional practice trials; see Palmer et al. 2026), as the overall task format remains unchanged, data from both apps were merged. For participants with multiple completions (e.g., due to repeat visits or technical issues), the first available completion was taken provided 17 valid trials were available. This cutoff was chosen based on prior research indicating that 15–20 trials offer a suitable balance between task length and accuracy (Plans et al. 2021; Palmer et al. 2026; Todd et al. 2024). Of 657 participants, 53 were excluded due to pregnancy at the time of testing. 67 participants' data was excluded due to completion of less than 17 valid trials. We also excluded 11 participants who, due to a technical problem with the application, completed additional trials. The final sample comprised of 526 participants (M_Age =_ 29.2, SD_Age_ = 9.18).^3^

### 1.3 Materials

All participants completed the Phase Adjustment Task (PAT) via a purpose‐built smartphone application described in Plans et al. (2021). During the task, participants placed their finger over their smartphone's camera and flash, allowing for heartbeat detection via photoplethysmography (PPG). The application used the device's available camera hardware and automatically selected the highest achievable sampling (frame) rate supported by the device to maximize temporal resolution of the optical signal. Following a three‐minute baseline period used to record resting heart rate, participants were presented with instructions for completing the task.

Heartbeat detection was implemented using an automated peak‐detection algorithm applied to the PPG waveform. To reduce noise, several physiologically motivated thresholds were applied. Detected inter‐beat intervals (IBIs) shorter than 400 ms or longer than 1.8 s were discarded due to implausible heart rates. In addition, any IBI differing by more than 30% from the previous valid IBI was rejected to minimize the influence of transient artifacts or ectopic heartbeats. When an IBI was rejected, the algorithm accepted the next detected period that satisfied the above criteria. The task did not impose explicit constraints related to heart rate beyond the physiological thresholds described above. As the smartphone camera utilizes white light for the PPG (rather than green/red light that has shown issues for darker skin tones; Bent et al. 2020; Chari et al. 2020), the task is suitable for all regardless of skin tone. While PPG can be affected by skin temperature (Khan et al. 2015; Jeong et al. 2014), as described in Palmer et al. (2026), when the heartbeat recording is disrupted, the presentation of tones is paused until a satisfactory reading can be made that meets the aforementioned criteria.

In the PAT, participants are presented with a series of auditory tones that are at the same frequency as, but out of phase with, their actual heartbeats. On each trial, the starting delay is randomized. Participants adjust a virtual dial to advance or delay the tones until they perceive them to be synchronous with their heartbeat. Auditory stimuli consisted of brief pre‐recorded tones 30 ms in length presented via the smartphone speaker. Tone presentation was triggered immediately following PPG heartbeat detection, with the addition of the randomized starting delay or the participant's currently selected delay. Each participant completed either two (original application) or four (new application) practice trials, followed by 20 main experimental trials. A subset of participants (*n* = 282) also completed a structurally identical screener task prior to the PAT (*n* = 111 completed the PAT and screener on the same day). In this task, participants are presented with two auditory tones and asked to adjust the virtual dial until they perceive these to be synchronous (Plans et al. 2021; Study 2). Unbeknownst to participants, one tone was triggered by their heartbeat, so that heart rate and heart rate variability (HRV) were matched across the PAT and screener task on an individual participant basis. This task was included to give confidence that any failure on the PAT was not due to difficulties with timing ability, lack of attention, motivation, or wider cognitive impairment, allowing accurate determination of the proportion of participants performing above and at chance to quantify the proportion of phase‐based and delay‐based responders.

## 2 Analysis

PAT data were exported in JSON format and processed using R Studio (v2023.12.1). Data were cleaned and organized using the *tidyr* package. Practice trials were excluded from all analyses. Quality filters removed trials with zero dial movements or durations spanning four or fewer heartbeats. Participants with fewer than 17 valid trials (out of 20) were excluded from further analysis. To standardize across participants, only the first 17 valid trials were retained for each participant to compute consistency and engagement metrics. IBI variability over trials was calculated as the standard deviation (SD) of the final sequence median IBI across trials, where the final sequence is determined by the sequence of dial positions within 5 degrees of the final position. It should be noted that unlike standard HRV, which indexes beat‐to‐beat variability over continuous recordings, IBI variability over trials captures variability in heart rate at the specific point at which participants submit their heartbeat‐tone synchrony judgment. As the phase‐based scoring approach is mildly influenced by extreme IBI variability over trials, we checked for participants whose IBI variability over trials exceeded the recommended threshold of 0.36 s SD (none were found, and therefore no data was excluded; see Palmer et al. 2026). Baseline heart rate data were cleaned by first remove missing or null values, then filtering IBIs to retain only physiologically plausible values within a predefined range (0.5–1.5 s). From these, the longest continuous segment of valid IBIs was identified using run‐length encoding, and only this segment was retained for further analysis. To calculate mean HR, at least 2 s of valid baseline data were required. To calculate HRV metrics, participants were required to meet a minimum data threshold of 30 s of valid baseline data; those who did not meet this criterion were retained in the dataset but flagged as having insufficient baseline HRV data, and baseline HRV metrics were not computed for these cases.

### 2.1 Task Scoring

As described in Palmer et al. 2026, PAT and screener data were analyzed using two approaches. In both, consistency was defined using delay values as periodic functions of the heartbeat period, calculated as: $\text{consistency}\;\left(d,p\right)=\frac{1}{n}\mathrm{mod}\left(\sum_{j=1}^{n}e^{i2\pi \frac{d_{j}}{p_{j}}}\right)$, where *d* is the delay and *p* is the heartbeat period.

#### 2.1.1 Phase‐Based

To calculate phase‐based consistency, we expressed delays as phase angles relative to each trial's median inter‐beat interval (IBI) at the final dial position (*p*). In other words, delays are interpreted as a proportion of the trial IBI. To determine whether performance is above chance, a group‐level cutoff was derived from 100,000 simulations of random delay sequences and randomly sampled heartbeat periods (0.5–1.5 s). The 95th percentile of this null distribution serves as the threshold for classifying responses as non‐random (interoceptive).

#### 2.1.2 Delay‐Based (Fixed‐Period)

To calculate delay‐based consistency, fixed‐period scoring was utilized. Here, all delays are interpreted as an absolute value using a constant, participant‐specific period *p*, calculated as the participant's overall median of the final sequence median IBIs. Because individual variability in IBI affects the chance distribution under this model (see Palmer et al. 2026), participant‐specific thresholds are generated from 10,000 simulated delay sequences per participant. The 95th percentile of each individual's null distribution is used as the cutoff for classifying their responses as interoceptive.

#### 2.1.3 Combined Classification

Participants are classified as interoceptive if they score above the chance threshold under either scoring method. This results in three distinct interoceptive profiles: (1) *Delay‐based interoceptive* (consistent absolute timing across trials); (2) *Phase‐based interoceptive* (consistent proportional timing within each cardiac cycle); and (3) *Ambiguous interoceptive* (consistent under both frameworks). Notably, classification under both models should not be interpreted as evidence of superior interoception. Rather, it likely reflects stable heart rates that render phase‐ and delay‐based responses indistinguishable (Palmer et al. 2026).

## 3 Results

Row a in Table 1 presents the results from the full sample analysis. As can be seen, 14.4% (*N* = 76/526) of participants performed above chance and were classified as interoceptive. Of the 76 participants classified as interoceptive, 21.1% (*N* = 16/76) were classified as such only using the phase‐based method (row c). As expected, IBI variability over trials was significantly lower in the ambiguous group (Median = 0.06, IQR = 0.06) compared to the participants classified under either phase or delay (Median = 0.16, IQR = 0.13; *U* = 231.50, *p* < 0.001), suggesting that low IBI variability over trials renders phase‐ and delay‐based responses indistinguishable.

**TABLE 1:** Analysis classifications in the (a) full unscreened sample and (b) screened sample. (c) Proportion of interoceptive participants classified under each analysis in the full sample.

	Interoceptive (phase)	Interoceptive (delay)	Interoceptive (ambiguous)	Non‐interoceptive
a	16 (3.0%)	15 (2.9%)	45 (8.6%)	450 (85.6%)
b	11 (3.9%)	10 (3.5%)	27 (9.6%)	234 (83%)
c	21.1%	19.7%	59.2%	

After restricting the sample to individuals who had completed the screener task and performed above chance (under any method), 282 participants remained (Table 1, row b). Of these, 17% (*N* = 48/282) were classified as interoceptive. Of the 48 participants classified as interoceptive, 22.9% (*N* = 11/48) were classified using only the phase‐based method. To estimate the false positive rate associated with the PAT 2.0 classification procedure, we conducted simulation analyses that reproduced the full scoring pipeline while incorporating sample‐specific cardiac dynamics. Median IBIs were extracted from a sample dataset and used to generate simulated participants by sampling these values with replacement. For each simulated participant, 17 trials were generated to match the minimum number of trials required for analysis. For each trial, delays were sampled uniformly within the allowable range $\left[-p/2,p/2\right]$, where p was the participant‐specific median IBI. Phase‐consistency and fixed‐period similarity scores were computed using the same circular similarity statistic implemented in the PAT 2.0 analysis. Phase‐based thresholds were derived from 100,000 simulated null datasets, while fixed‐period thresholds were derived from 10,000 participant‐specific simulations using the participant's median IBI. Simulated participants were then classified according to the same phase and fixed‐period criteria applied to the empirical data. This procedure allowed estimation of the expected false positive rates for phase‐only, delay‐only, double‐positive, and overall interoceptive classifications under the null hypothesis of random responding. Under the null, phase‐only and delay‐only classifications were exceedingly rare (expected rates of ~0.02% and ~0.3%, respectively), whereas ambiguous (both) classifications occurred at a rate of ~4.55%. Applied to the present sample (*N* = 526), this corresponds to approximately 1 phase‐only participant, fewer than 1 delay‐only participant, and ~24 ambiguous participants expected by chance. In contrast, the observed data contained 16 phase‐only, 15 delay‐only, and 45 ambiguous participants. This simulation revealed that any false positives are vastly over‐represented in the ambiguous dataset, with few to none expected in either the phase or delay groups.

**FIGURE 1:**
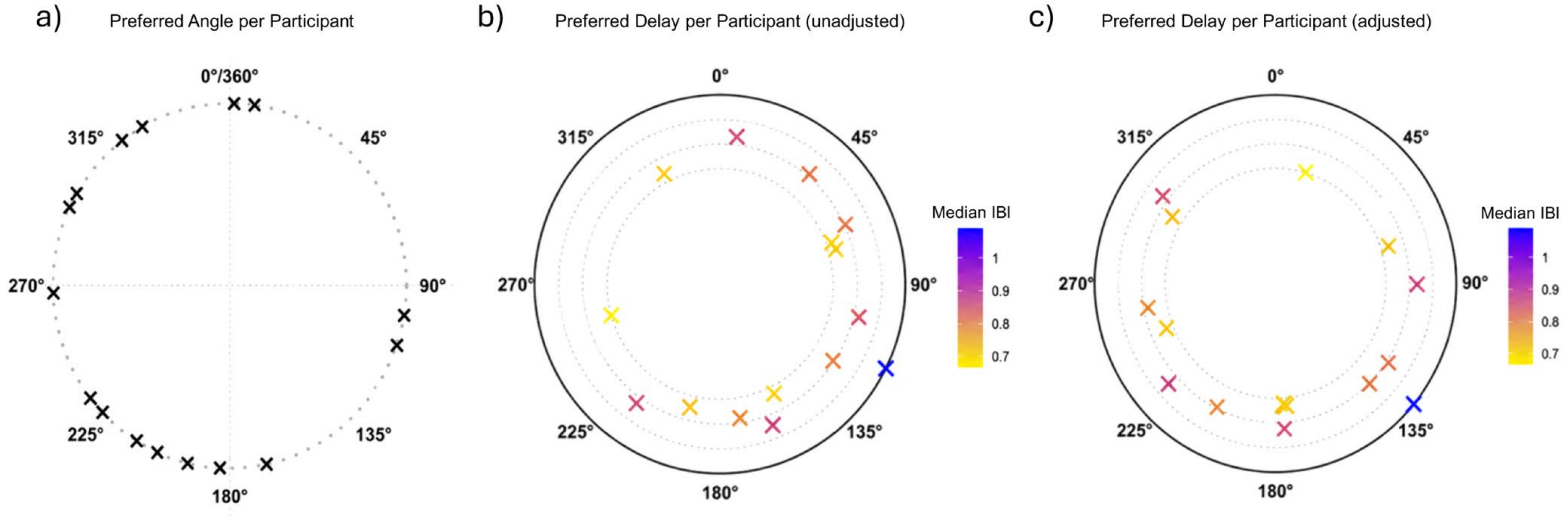
(a) Preferred phases for *N* = 16 phase‐only participants, represented as angles on a circle. (b) Preferred delays for *N* = 15 delay‐only participants. Delays are represented as angles on IBI‐scaled circles (smallest to largest circles represent the smallest to largest median IBIs taken from the median values of the final sequence IBI on each trial, respectively). (c) Preferred delays for *N* = 15 delay only participants, adjusted to account for pulse arrival time (PTT; estimated at 200 ms; Allen and Murray 2003). Each participant's preferred delay was scaled relative to their median IBIs taken from the median values of the final sequence IBI on each trial. As in figure (b), delays are represented as angles on IBI‐scaled circles.

The distribution of selections for those classified as phase‐based or delay‐based responders is presented in Figure 1. Preferred phases and delays were calculated using circular averaging: each trial's delay was converted to a phase angle (in radians) based on the participant's final sequence median IBI during the trial, then transformed into Cartesian coordinates. For each participant, the mean vector was computed by summing the x and y components across trials, and the preferred angle was derived using the arctangent of the summed coordinates. As can be seen, for both phase‐based (Figure 1a) and delay‐based (Figure 1b) responders, selections were distributed across the entire heartbeat cycle. For delay‐based responders, this was true even after approximately accounting for PTT to the finger (estimated at 200 ms; Allen and Murray 2003; Figure 1c).^4^ Individual participant trial‐by‐trial response data are provided in the Supporting Information (Figures S1 and S2), alongside analysis of physiological and engagement metrics (Figure S3) and similarity scores for each participant as a function of classification (Figure S4). Although valid baseline data were not available for all participants, where available only heart rate significantly differed between phase‐based and delay‐based responders and was significantly higher for delay‐based responders (M_phase_ = 71.32, M_delay_ = 83.29). No other significant differences in physiological or engagement metrics were observed between delay‐based and phase‐based responders.

## 4 Discussion

This study aimed to elucidate whether a subset of individuals perceive heartbeat‐tone synchrony at a consistent phase of the cardiac cycle, rather than at a fixed delay after the R‐wave. As hypothesized, we found a subset of participants who displayed non‐random responding that was consistent with a phase‐based selection strategy. Among those who exhibited non‐random responding on the PAT, 21.1% (*N* = 16/76) were classified solely as phase‐based responders. Simulations suggested the phase‐based responders were extremely unlikely to be false positives, and the observed proportion probably underestimates the true proportion of individuals making phase‐based responses. Indeed, the highest proportion of interoceptive individuals (59.2%; *N* = 45/76) were classified as exhibiting non‐random response patterns under both delay‐based and phase‐based scoring methods. This is likely due to low IBI variability over trials making delay‐based and phase‐based responses indistinguishable. Importantly, when examining phase‐based and delay‐based responses on an individual participant basis, timings varied considerably across participants, spanning the entire cardiac cycle. Overall, these results directly challenge the prevailing assumption that heartbeat sensations are uniformly tied to a fixed delay after the R‐wave and demonstrate considerable variability in the perception of heartbeat‐tone synchrony for those using both phase‐based and delay‐based strategies.

These findings have important implications for the analysis and interpretation of data across a range of cardiac interoceptive accuracy tasks. Indeed, they suggest that individuals selecting multiple delays on tasks such as the MCS and 6AFC may reflect the presence of phase‐based responders, as well as potentially the use of different bodily locations or differences in the duration of the heartbeat stimulus vs. presented tones producing trace effects as previously hypothesized (Brener and Ring 2016) and described above. Such possibilities raise concerns regarding the use of IQR and beat‐to‐tap consistency for the analysis of the MCS/6AFC and HTT, respectively, and have implications for other tasks that infer accuracy solely from the consistency of participants' selected delays, including the original (but not revised) scoring of the PAT (e.g., Moffatt et al. 2024; Plans et al. 2021; Ring and Brener 2018; Ruisch et al. 2023; Smith et al. 2021). While the IQR and beat‐to‐tap consistency capture the spread and consistency of delay responses, they do not appropriately capture the spread and consistency of phase‐based responses. For phase‐based responders with high IBI variability, delay selections will likely vary as a function of natural fluctuations in heart rate during the task. Such response patterns would result in a larger IQR and lower beat‐to‐tap consistency, suggesting imprecise responding when responses may be extremely precise relative to cardiac phase. This is less likely to be the case when utilizing other scoring approaches. Indeed, where χ^2^ tests are used to capture non‐random response patterns for the MCS/6AFC, it is likely that fewer participants making phase‐based responses would be classified as non‐interoceptive. This is because χ^2^ based scoring does not assume that single‐delay selections represent more consistent responding, and thus so long as preferred phases are represented across the delays used to present stimuli, non‐random responders are more likely to be correctly identified.

Although the use of a binary classification system (interoceptive or not interoceptive) may mitigate the likelihood that interoceptive individuals who perceive their heartbeat at a precise phase of their cardiac cycle may be incorrectly classified where heart rate varies over trials, even a binary classification approach for multi‐delay tasks may still misclassify individuals whose preferred phase (or preferred delays for those utilizing a delay‐based strategy) are not represented by the delays used to present stimuli. Indeed, when examining the distribution of phase‐based and delay‐based responses, we observed that selections ranged across the entire cardiac cycle, suggesting that not all individuals perceive heartbeat‐tone synchrony at a delay or phase that would always correspond with the typical delays used to present stimuli on tasks such as the MCS/6AFC (e.g., 0–500 ms following the R‐wave).

While it is unclear why some individuals display non‐random response patterns that do not fall into the expected range based on previous data (e.g., 0–400 ms; Clemens 1984; Yates et al. 1985; Betka et al. 2020), to our knowledge no study has examined the proportion of synchronous responses across a range of delays beyond 0‐500 ms following the R‐wave. As the PAT allows responses across the entire cardiac cycle for all participants (of course, a range of 0‐500 ms would span the entire cardiac cycle for an individual with a heart rate of 120 bpm), one possibility is that distributed response patterns reflect the fact that some participants may synchronize using a tone‐heartbeat strategy, rather than a heartbeat‐tone strategy (see also Smith et al. 2020, 2021). Such a possibility may be more likely where heart rate is faster and HRV is lower, as stimuli presented at later delays on tasks such as the MCS/6AFC may coincide with the next heartbeat cycle. An alternative possibility is that requiring participants to judge whether stimuli are synchronous or asynchronous with heartbeats (as in the MCS/6AFC HDT) constrains responses. Thus, pronounced individual differences in the perception of heartbeat‐tone synchrony may emerge only where individuals are free to report the perception of heartbeat‐tone synchrony at any point in the cardiac cycle, as is the case in the PAT. It is therefore possible that previously reported consistency across participants on tasks such as the MCS/6AFC HDT may partly reflect task constraints rather than true consistency across participants. Another possibility is physiological. Indeed, there are several possible physiological explanations for both phase‐based response patterns and distributed delays. One explanation may be differential sensitivity to baroreceptor firing, which varies across the cardiac cycle (such that firing decreases during diastole; Katayama et al. 2018). Alternatively, individuals may be perceiving peripheral mechanoreceptor input associated with vascular distension (e.g., in the neck or fingers), that begins during systole but continues into diastole (e.g., Levick 2010; Caro et al. 2011) and has been implicated in heartbeat perception processes (e.g., Brener and Ring 2016; Khalsa et al. 2009). If some individuals are sensitive not to the onset of vascular distension but to later mechanical changes such as the release of pressure (the “off” signal) or slower vessel recoil during diastole, then their perception of heartbeat‐tone synchrony may occur later in the cardiac cycle and at a consistent proportion of the cardiac cycle rather than at a fixed delay. Indeed, somatosensory processing of stimuli has been shown to decrease during systole, potentially as a result of baroreceptor‐driven inhibitory influences on cortical excitability (Duschek et al. 2013; Motyka et al. 2019; Al et al. 2020, 2021). This cardiac‐phase–dependent gating could result in masking of interoceptive cardiac signals during systole, which instead may be preferentially detected during diastole, when baroreceptor activity is reduced and sensory processing is relatively facilitated. Notably, although our results revealed differences in baseline heart rate data between phase‐only and delay‐only responders (see S3; though this was in small samples of 12 and 11 participants, respectively), with delay‐based responders exhibiting higher heart rates (i.e., shorter IBIs), we believe this is unlikely to reflect that individuals with longer IBIs are inherently more likely to adopt a phase‐based strategy; rather, longer IBIs provide a greater window over which variability in absolute delays can arise while preserving consistency in relative phase, making phase‐based response patterns more readily detectable using the present methods.

Although the above proposals are inevitably speculative and further work integrating physiological measures will be required to test these hypotheses, distributed response patterns and evidence consistent with the presence of phase‐based responders highlight the importance of utilizing tasks that span the entire cardiac cycle for all participants and thus do not make assumptions about the timing at which individuals perceive a tone to be synchronous with their heartbeat (i.e., at a certain delay following the R wave or at systole; Brener and Ring 2016; Whitehead et al. 1977). Indeed, it is clear from these data that there may be a subset of individuals who exhibit non‐random response patterns that are not well captured by traditional tasks and delay‐based scoring methods. Although binary classification approaches for tasks such as the MCS/6AFC tasks are likely to identify non‐random response patterns within the common range of delays used to present stimuli, it is unclear how many individuals would be misclassified where preferred delays/phases fall outside the common range. While the exact proportion of individuals who have preferred delays/phases that are not represented by tasks such as the MCS/6AFC remains unknown, it is likely that where preferred phases/delays are not consistently represented (either because the preferred delay/phase is not presented or because high IBI variability over trials means that the preferred phase is not consistently presented) responses will be less consistent.

While we have focused on tasks presenting stimuli at multiple delays above, it is worth noting that evidence of distributed response patterns on the PAT suggests that any method that requires a response within a particular delay window, or at a particular phase, of the cardiac cycle could potentially miss non‐random responding in certain circumstances. Indeed, although the Bayesian analysis method for the HTT provides a broader response window (e.g., 350–650 ms or R‐peak to 50% of the IBI; Körmendi et al. 2022; Smith et al. 2020) which may better accommodate IBI variability and phase‐based response patterns, this approach for scoring the HTT may still misclassify participants whose preferred phase or delay falls near the boundary or outside of this predefined window. The extent to which this occurs for current approaches for the analysis of the HTT and for the MCS/6AFC cannot be determined from these data. Indeed, where heartbeat is recorded from a distal bodily location (e.g., the finger, as in the current implementation of the PAT, or the ear in instances of the HTT; see Allen and Murray 2003; Smith et al. 2020), PTT must be approximated in order to estimate selected delays/phases, and such approximations are unlikely to be accurate for all (Budiman et al. 2022; Chan et al. 2007; Wibmer et al. 2014). To accurately approximate the likelihood of misclassification under other methods, ECG implementation of the PAT would be required to remove assumptions surrounding PTT, thus enabling the exact delays and phases of the cardiac cycle to be determined. Nevertheless, as preferred delays/phases were distributed across the entire cardiac cycle, the current data do suggest that misclassification may occur in some cases.

Overall, our findings suggest that to capture accurately non‐random responses in tasks examining heartbeat‐tone synchrony (or synchrony between heartbeats and motor responses), tasks and associated analytic approaches should: (1) enable responses across the entire cardiac cycle for all individuals; (2) capture all patterns of non‐random responding (including phase‐based response patterns); and (3) avoid assumptions regarding the timing at which individuals perceive heartbeat‐tone synchrony. Although novel analyses of the HTT and the MCS/6AFC achieve some of these aims (and represent notable improvements on the 2AFC HDT; see Desmedt et al. 2023), to our knowledge only PAT 2.0 achieves all of these aims. While this is achieved using a classification‐based scoring system (conceptually similar to the χ^2^ test used for the MCS; Brener and Ring 2016), the use of such a system is essential to capture both phase‐based and delay‐based non‐random response patterns. Although a binary scoring system incurs a notable loss of statistical power in comparison to a continuous score (Cohen 1983), the gain in classification accuracy (compared to measures such as the IQR or beat‐to‐tap consistency when used with phase‐based responders with high IBI variability) likely justifies this trade‐off. Indeed, where continuous scores are not meaningful because the assumptions made do not hold for all participants, the apparent gain in statistical power is illusory. Importantly, implementation of the PAT via a smartphone application facilitates large‐scale remote administration, which will enable the collection of adequately large samples to offset any potential loss of power.

In summary, these data challenge the prevailing assumption that heartbeat‐tone synchrony occurs at a fixed delay post R‐wave, demonstrating that a notable subset of at least ~21% (*N* = 16/76) of interoceptive individuals perceive synchrony at a particular phase of the cardiac cycle. While the differentiation between delay‐based and phase‐based responding should not be interpreted as implying functional superiority of either mode of perception, this evidence highlights a potential limitation of existing interoceptive measures, which assume that heartbeat perception occurs at a fixed temporal delay for all individuals, and provides an initial estimate of the proportion of individuals that may be misclassified by existing measures. This evidence may also partly explain multiple delay selections on the MCS/6AFC, and highlight a need for caution when utilizing the IQR as the sole outcome measure when scoring this task. Importantly, further research is required to determine the exact proportion of individuals who exhibit phase‐based response patterns as the current estimate is likely to be an underestimation of the true number of phase‐based responders. Such work should examine performance under conditions where heart rate varies over trials (e.g., due to exercise, caffeine etc.) to better distinguish between delay‐based and phase‐based responders. As well as evidence of phase‐based responding, for both delay‐based and phase‐based responders, a wide distribution of preferred delays/phases was observed across participants which spanned the entire cardiac cycle. While further work using ECG methods is required to accurately estimate preferred delays/phases across individuals, such work is essential for estimating the likelihood of false negatives on other tests of cardiac interoceptive accuracy. Overall, these data highlight the importance of capturing all types of non‐random response patterns on measures of cardiac interoceptive accuracy to accurately quantify individual differences in interoception.

## Author Contributions

**Jennifer Murphy:** writing – review and editing, conceptualization, data curation, supervision, formal analysis. **Jane Aspell:** writing – review and editing, data curation. **Geoffrey Bird:** writing – review and editing, conceptualization, supervision, formal analysis, data curation. **Jennifer Todd:** writing – review and editing, data curation. **Davide Morelli:** writing – review and editing. **Ren Palmer:** writing – original draft, writing – review and editing, formal analysis, conceptualization, data curation, methodology. **David Plans:** writing – review and editing, data curation. **Mateo Leganes:** writing – review and editing, data curation.

## Funding

JM is supported by a New Investigator Research Grant from the Medical Research Council (MR/X010295/1). JA is supported by a Pain Challenge Research Award from Versus Arthritis (22461).

## Conflicts of Interest

J.M. and G.B. have completed paid consultancy work for Healios for work on interoception.

## Supporting information


**Data S1:** Preferred angles for each phase‐only participant.
**Data S2:** Preferred delays for each delay‐only participant.
**Data S3:** Comparison of physiological and engagement metrics between delay‐ and phase‐based responders.
**Data S4:** Comparison between phase and delay scores in interoceptive participants.
**Data S5:** Datasets.

## Data Availability

The data that support the findings of this study are available from the corresponding author upon reasonable request.

## Endnotes

^1^
Note that ‘interoceptive’ or ‘non‐interoceptive’ here refers to the individual's ability to perceive their heartbeats at the point in time in which the task is completed. This alternative to ‘heartbeat perceiver’ has been used in numerous published papers and is used to describe individuals who exhibit above chance performance on a specific task at a specific point in time (e.g., Plans et al. 2021; Spooner, Bird, Clemente, et al. 2024; Spooner, Bird, Irigoras Izagirre, et al. 2024).

^2^
We refer to pulse arrival time as PTT for clarity to distinguish from the Phase Adjustment Task (PAT).

^3^
Note that demographic data was unavailable for a small subset of participants (*N* = 24); therefore, the demographic information presented reflects all available data.

^4^
Note that an adjustment of 200 ms would not be equivalent across participants because individuals differ in their IBIs, meaning that a fixed temporal shift corresponds to different relative proportions of the cardiac cycle for different participants.
