# Supplementary material for: Evidence for Cardiac Phase‐Linked Perception of Heartbeats
Source: Psychophysiology. 2026 Jun 25;63(6):e70346. doi: 10.1111/psyp.70346 (PMC13296266; doi:10.1111/psyp.70346)
Supplement: Supplementary file 1 — Data S1: Preferred angles for each phase‐only participant. Data S2: Preferred delays for each delay‐only participant. Data S3: Comparison of physiological and engagement metrics between delay‐ and phase‐based responders. Data S4: Comparison between phase and delay scores in interoceptive participants. Data S5: Datasets. [file PSYP-63-e70346-s001.docx]

**Supplement**

**S1: Preferred angles for each phase-only participant**

**
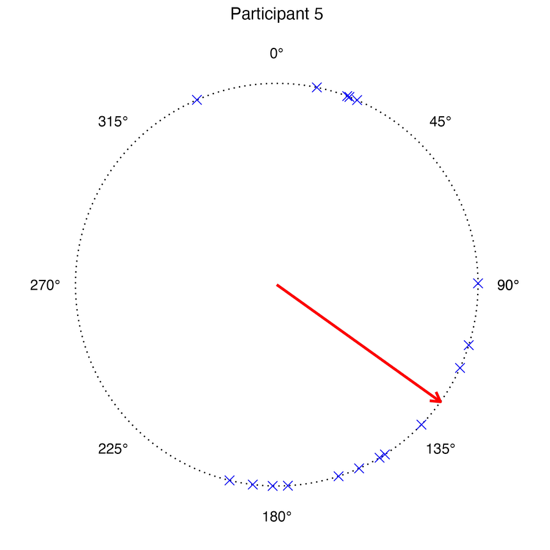

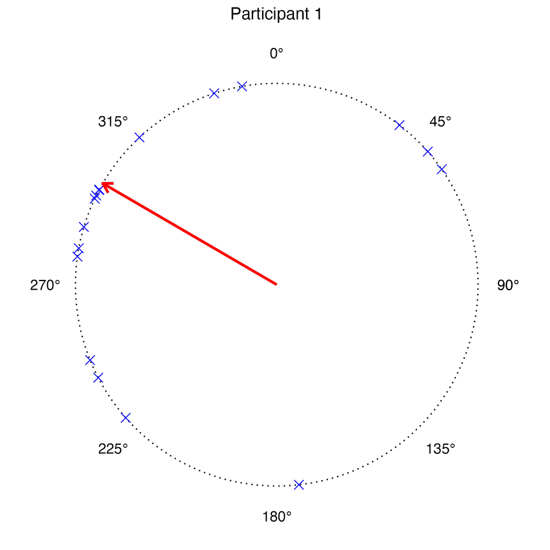

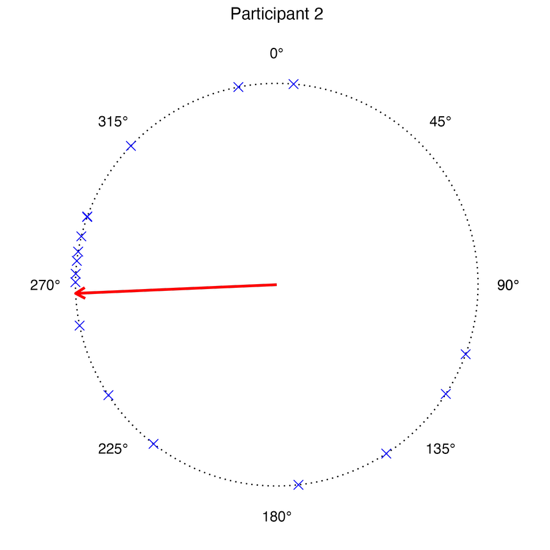

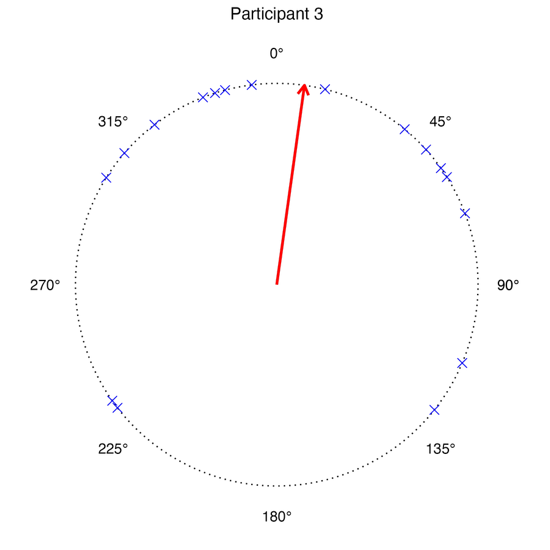

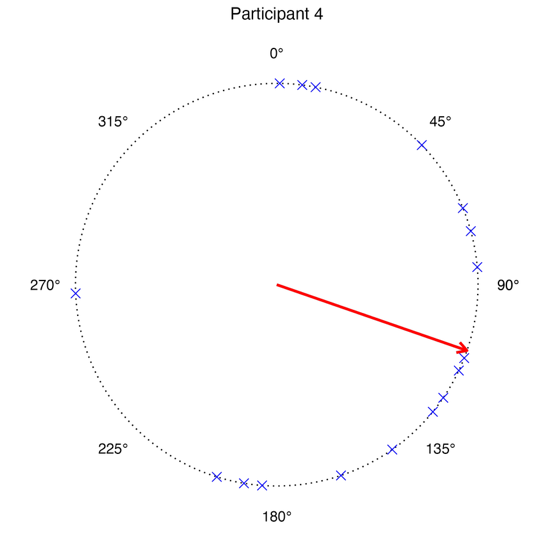

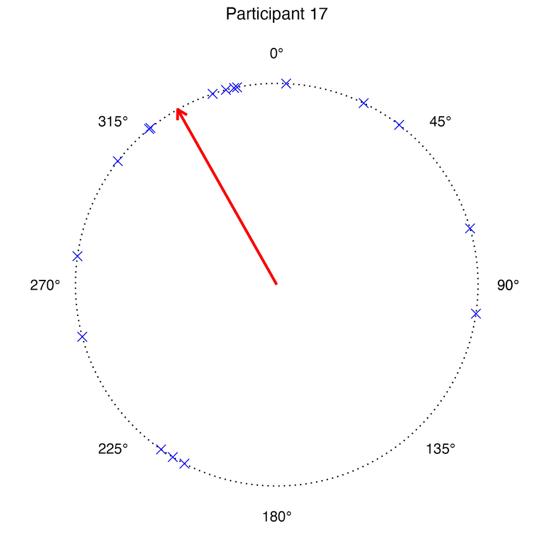

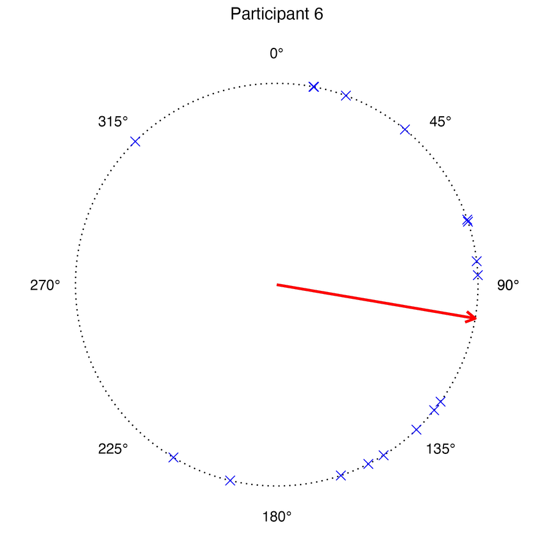

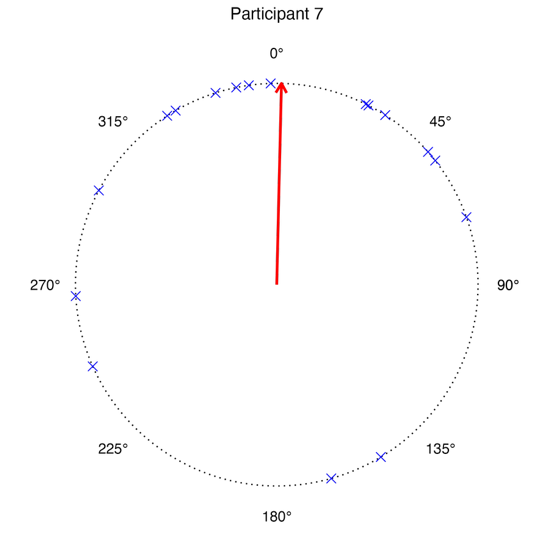

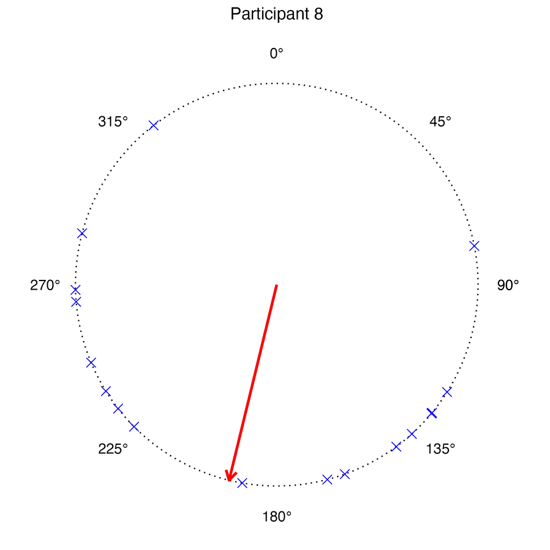

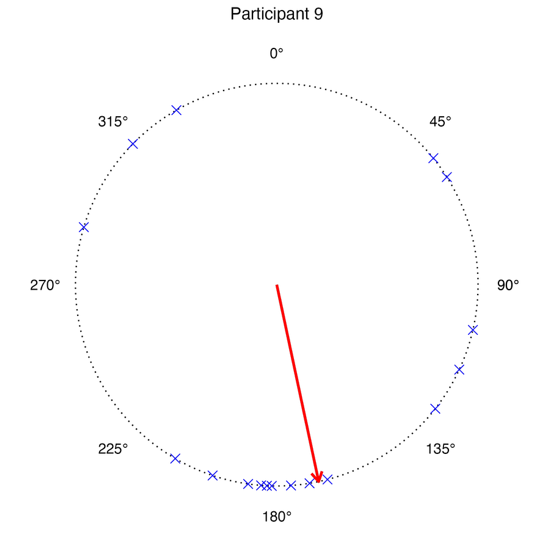

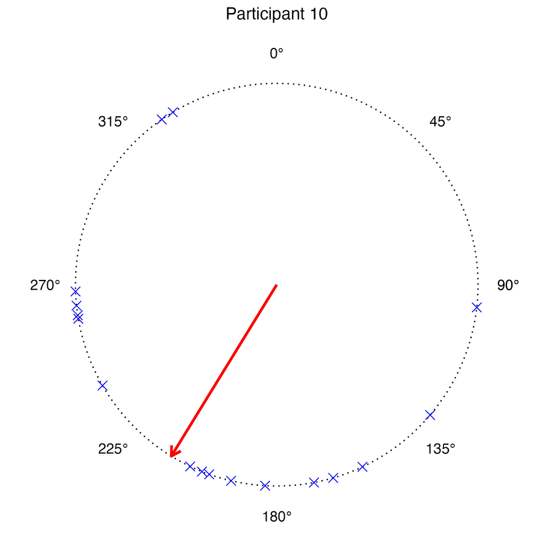

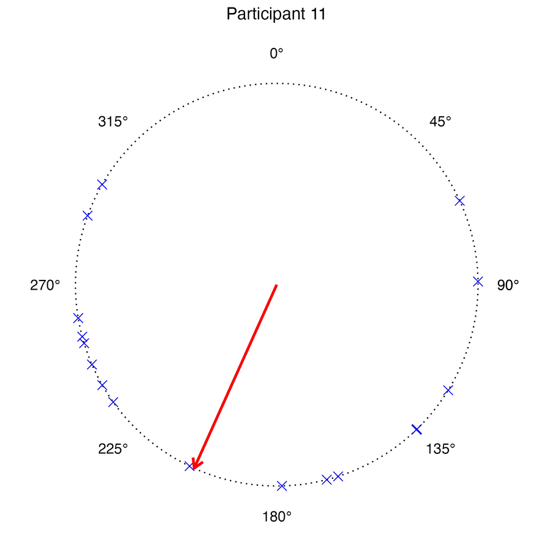

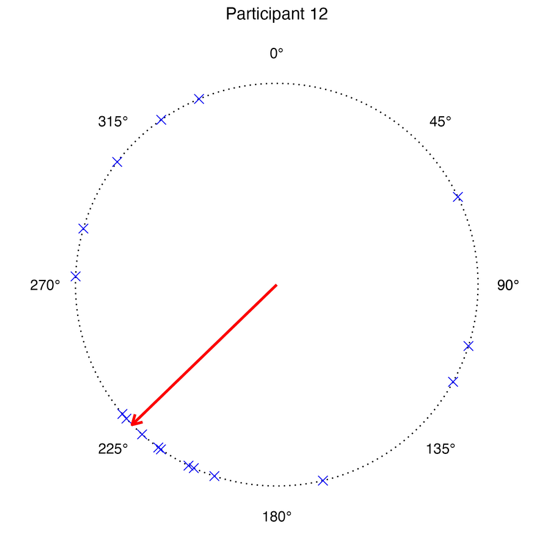

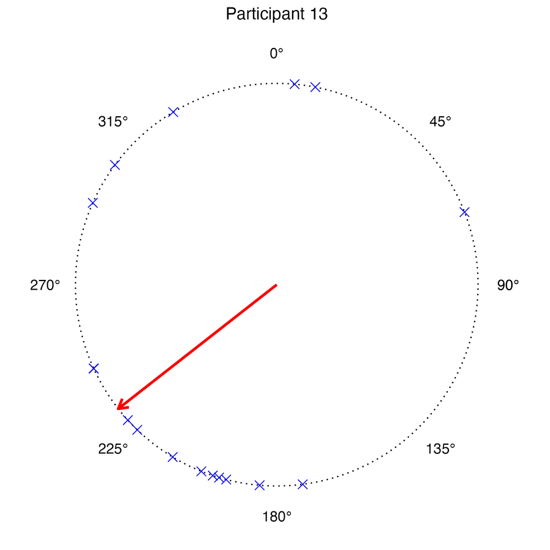

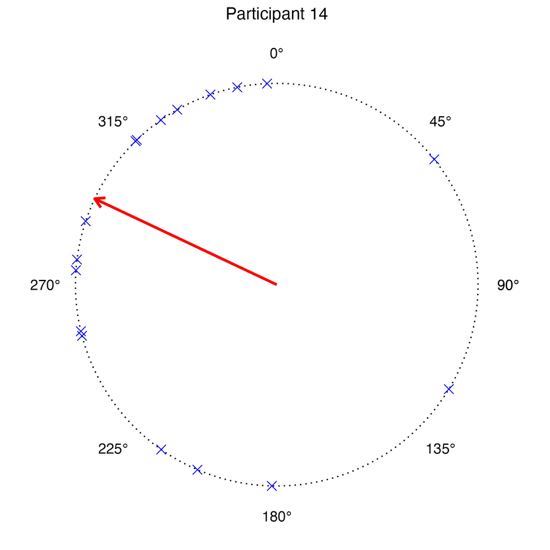

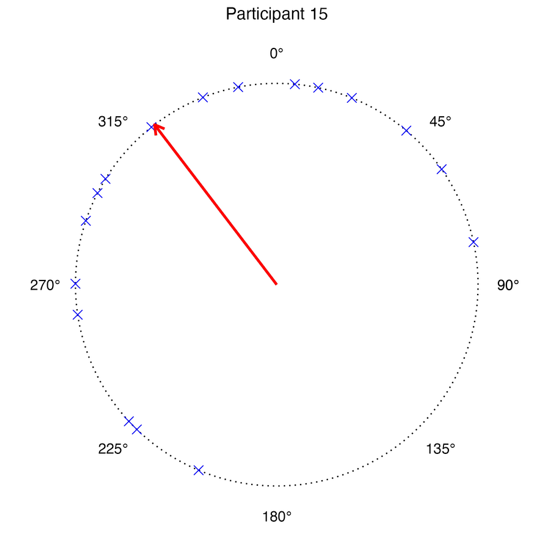

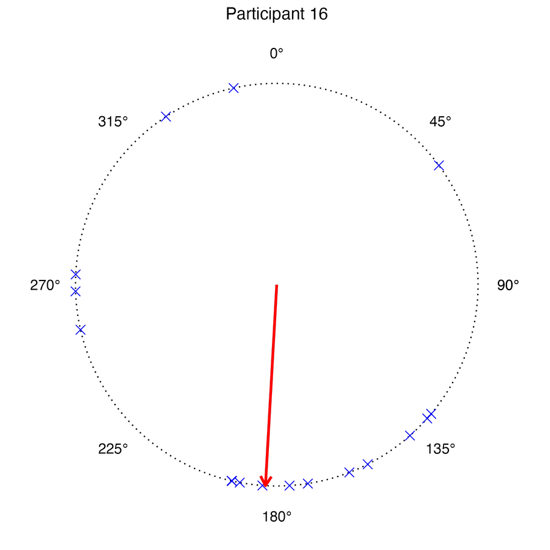
**

*Each subplot displays one participant’s trial-level phase responses (blue crosses) around the cardiac cycle, plotted on a circular scale (0–360°) representing the trial-by-trial median IBI, taken as the median value of the final sequence IBI. The red arrow indicates the participant’s* ***preferred phase****. These were calculated by converting each trial’s selected delay to a phase angle (based on the participant’s median IBI, taken as the median value of the final sequence IBI during the trial), then transforming this angle into Cartesian coordinates. The mean vector was computed by summing the x and y components across trials, and then the preferred angle was derived using the arctangent of the summed coordinates. Axes labels correspond to degrees around the cardiac cycle (with 0° at the top, increasing clockwise). The overall median IBI, calculated from the median values of the final sequence IBIs, and range of trial-by-trial median IBIs are labelled.*

**S2: Preferred delays for each delay-only participant**


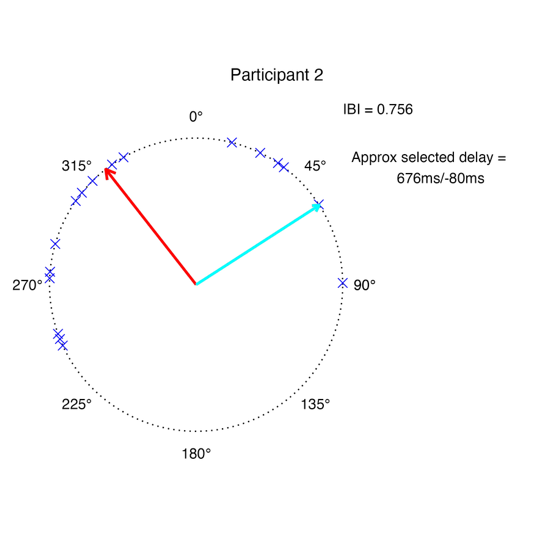


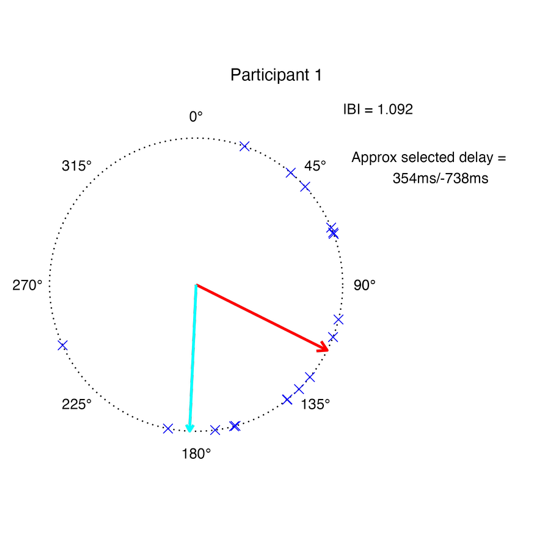

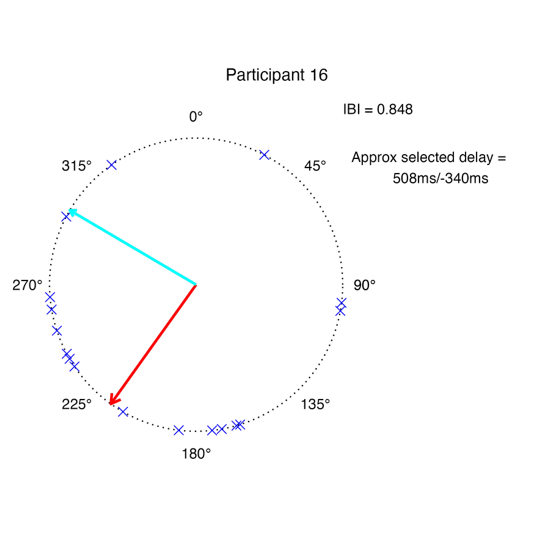

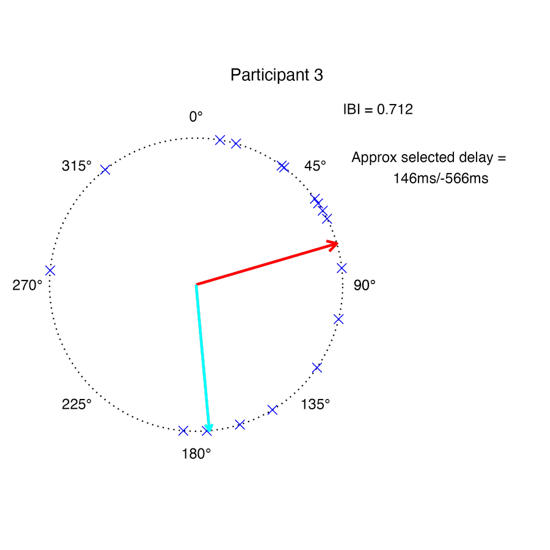

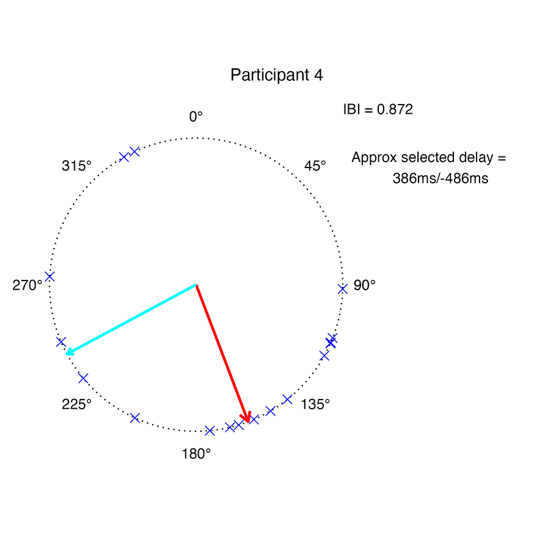

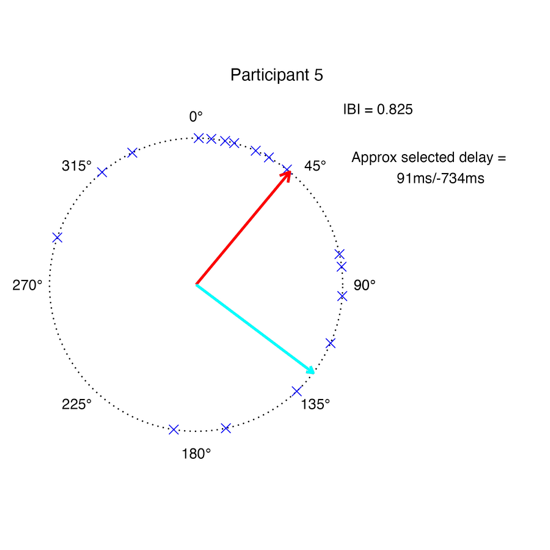

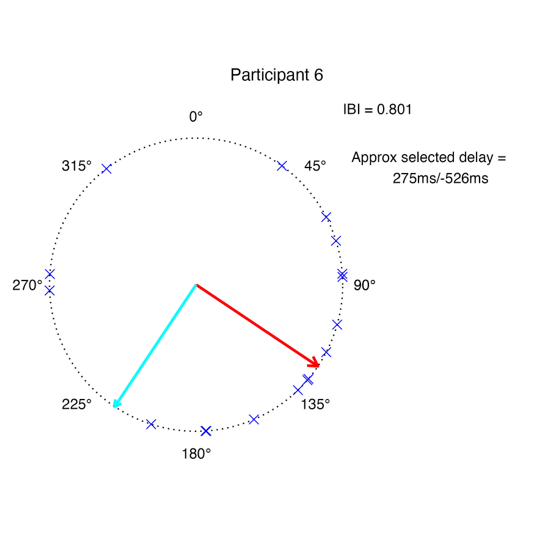

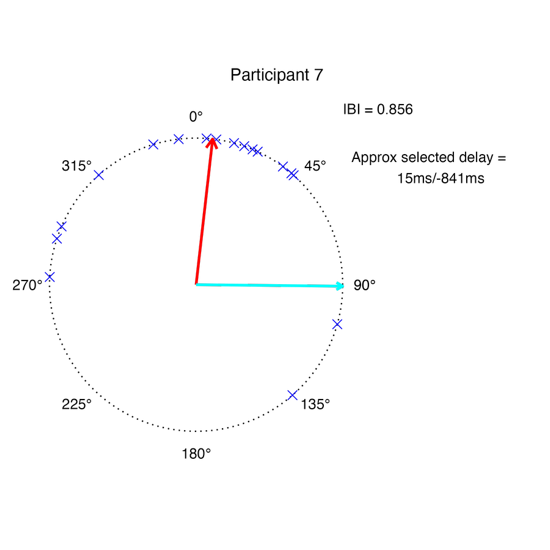

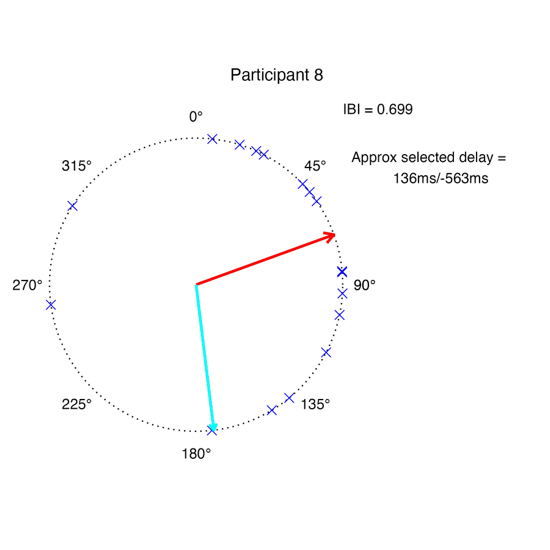

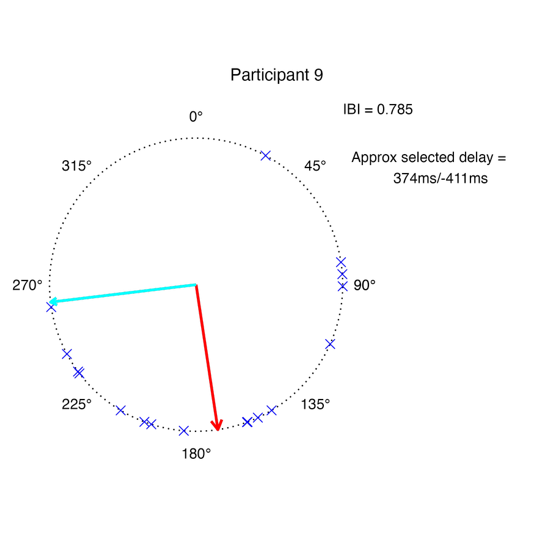

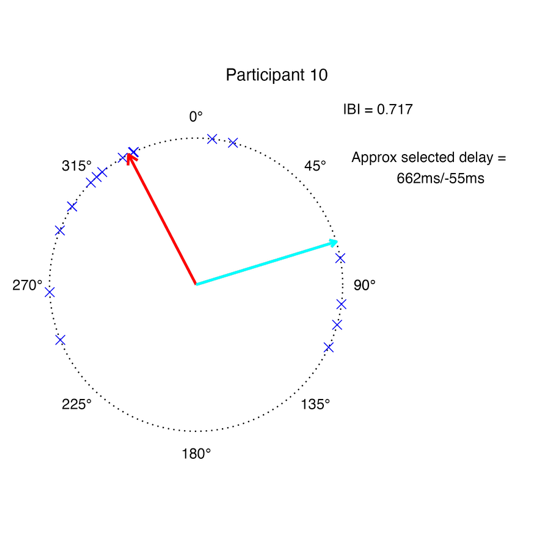

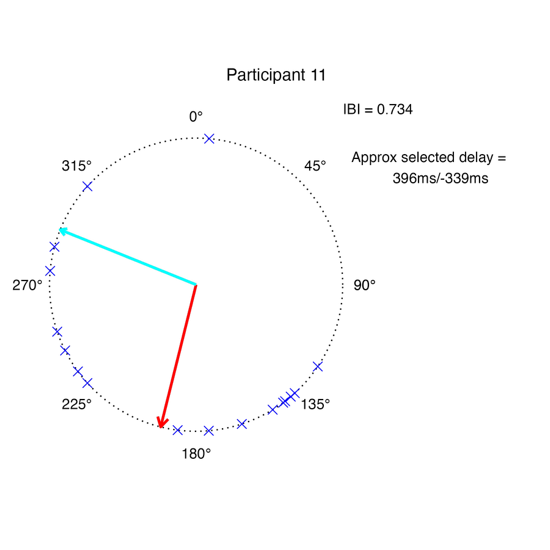

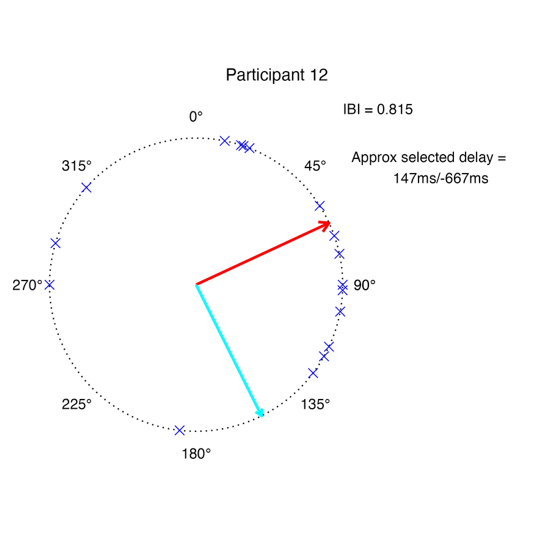

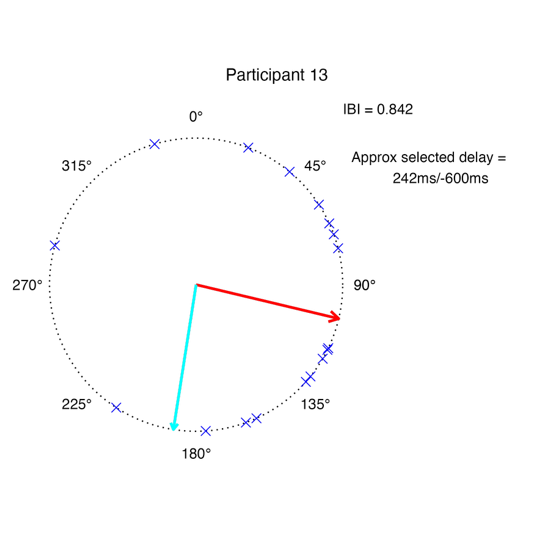

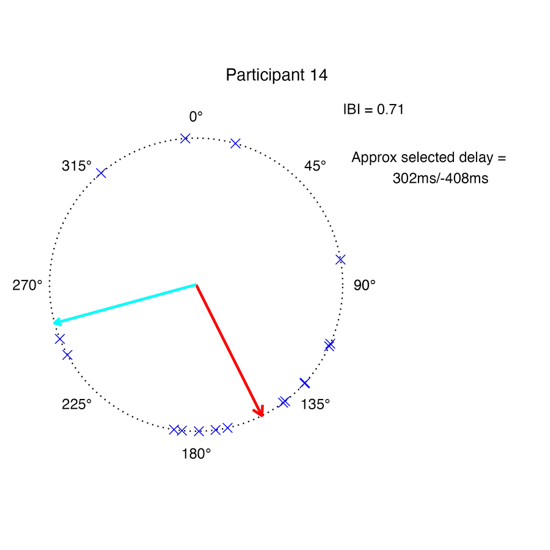

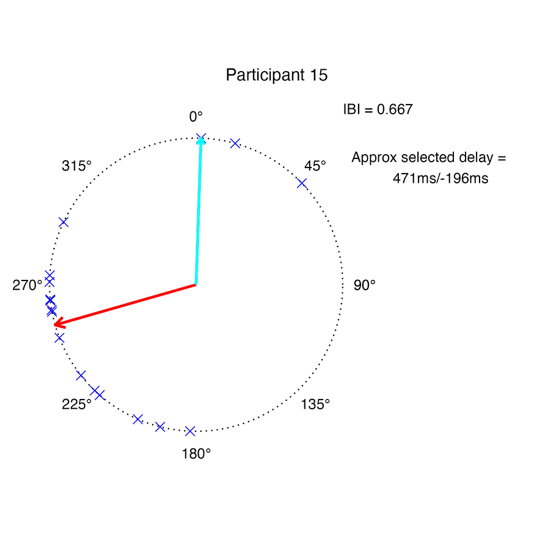


*Each subplot displays one participant’s trial-level delay responses (blue crosses) around the cardiac cycle, plotted on a circular scale (0–360°) representing the overall median IBI across trials, calculated from the median values of the final sequence IBIs. The red arrow indicates the participant’s* ***preferred delay.*** *These were calculated by converting each trial’s selected delay to a phase angle (based on the participant’s median IBI, taken as the median value of the final sequence IB during the trial), then transforming this angle into Cartesian coordinates. The mean vector was computed by summing the x and y components across trials, and then the preferred angle was derived using the arctangent of the summed coordinates. The cyan arrow indicates this preferred delay shifted forward by 200ms (to approximately account for pulse transit time; Allen & Murray, 2003). Approximate delay values (in milliseconds) are expressed as both time from heartbeat (e.g., 400ms) and time before the next heartbeat (e.g., –300ms). Each participant’s overall median IBI across trials (used for calculation of angles) is given. Axes labels correspond to degrees around the cardiac cycle (with 0° at the top, increasing clockwise).*

**S3: Comparison of physiological and engagement metrics between delay- and phase-based responders**

|  | **Delay-only (N=11)** | **Phase-only (N=12)** | **U** | **p** |  |
| --- | --- | --- | --- | --- | --- |
|  | **M(SD)** | **M(SD)** |  |  |  |
| **Total time on task (s)** | | 324.00 (122.95) | 388.20 (207.74) | 94.0 | 0.318 |
| **Mean time per trial (s)** | | 19.06 (7.23) | 22.84 (12.22) | 94.0 | 0.318 |
| **Mean engagement (dial turns) per trial** | | 23.45 (9.39) | 26.32 (15.98) | 117.5 | 0.937 |
| **Number of valid trials** | | 19.33 (0.72) | 18.75 (1.13) | 154.5 | 0.155 |
| **Resting heart rate (bpm)** | | 83.29 (9.87) | 71.32 (9.83) | 203.0 | **<.001** |
| **Heart rate variability (SDNN)** | | 73.88 (44.31) | 95.36 (46.99) | 44.0 | 0.190 |
| **Heart rate variability (RMSSD)** | | 64.58 (45.74) | 86.58 (42.05) | 36.0 | 0.069 |
| **Heart rate variability (pNN50)** | | 27.68 (18.51) | 41.95 (18.12) | 36.0 | 0.069 |

*Engagement metrics and heart rate data for delay-only and phase-only participants.^[[1]](#footnote-1)^ Group-level differences tested using Mann-Whitney U tests.*

**
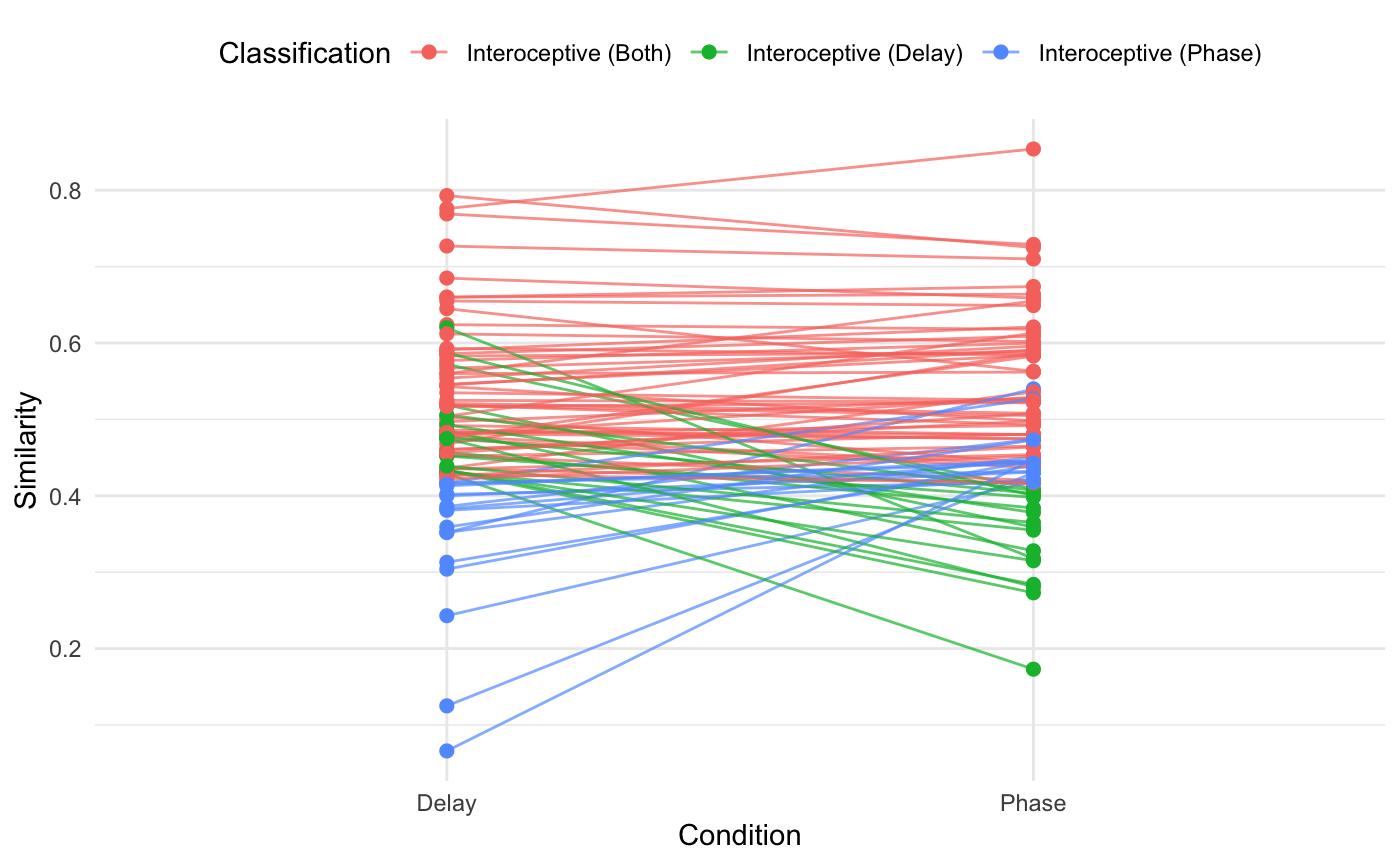
S4: Comparison between phase and delay scores in interoceptive participants**

*Figure 1: Individual participant similarity scores for phase-based and delay-based analyses. Each line represents one participant and connects their similarity scores across the two analysis methods. Lines are coloured according to response classification (phase-based vs. delay-based vs. ambiguous responders). This is just for illustration purposes – the use of continuous scores is not recommended, as the two are not directly comparable metrics, particularly when comparing between participants.* *Delay-based classifications are computed using individualised participant thresholds, due to the slight impact of IBI variability on scores (Palmer et al., under review)*

**S5: Datasets**

|  | Dataset 1 | Dataset 2 | Dataset 3 | Dataset 4 | Dataset 5 | Dataset 6 |
| --- | --- | --- | --- | --- | --- | --- |
| Final N | 102 | 148 | 64 | 12 | 92 | 108 |
| N with demographic data | 102 | 143 | 62 | 10 | 82 | 108 |
| N = female | 60 | 78 | 44 | 6 | 82 | 41 |
| Age (M and SD) | 23.51 (8.03) | 30.55 (9.17) | 27.41 (13.37) | 23.5 (3.31) | 40.35 (8.38) | 32.23 (6.55) |
| Data collection method | Laboratory | Remote | Laboratory | Remote | Remote | Remote |
| Recruitment and inclusion criteria | Aged 18–60 years. Normal/corrected hearing/vision | Aged 18–60 years. Access to an Apple iPhone meeting eligibility requirements. Normal/ corrected hearing/ vision | Aged 18–60 years. Normal/corrected hearing/vision | Aged 18–35 years; at least one night of heavy drinking in the past 30days (4 or 5 alcoholic drinks in less than 2h for females and males, respectively); consume more than 5 drinks per week; one hangover in past 30days; one blackout episode in the past 6months. No self-reported history of cardiovascular or mental health disorder. Access to an Apple iPhone meeting eligibility requirements | Aged 18–60 years. Normal/corrected hearing/vision. Access to an Apple iPhone meeting eligibility requirements. Normal/ corrected hearing/ vision | Aged 18–60 years. Normal/corrected hearing/vision. Access to an Apple iPhone meeting eligibility requirements. Normal/ corrected hearing/ vision |

1. Note that these values are after restricting to only participants who obtained valid baseline HRV data (at least 30 seconds of continuous baseline heart rate data), to allow for calculation of HRV metrics [↑](#footnote-ref-1)
